# *Bifidobacterium infantis* Maintains Genome Stability in Ulcerative Colitis *via* Regulating Anaphase-Promoting Complex Subunit 7

**DOI:** 10.3389/fmicb.2021.761113

**Published:** 2021-11-02

**Authors:** Taotao Han, Xiaomin Hu, Kemin Li, Di Zhang, Yan Zhang, Jingnan Li

**Affiliations:** ^1^Department of Gastroenterology, Peking Union Medical College Hospital, Peking Union Medical College, Chinese Academy of Medical Sciences, Beijing, China; ^2^Key Laboratory of Gut Microbiota Translational Medicine Research, Chinese Academy of Medical Sciences, Beijing, China; ^3^State Key Laboratory of Complex Severe and Rare Diseases, Department of Medical Research Center, Peking Union Medical College Hospital, Chinese Academy of Medical Sciences and Peking Union Medical College, Beijing, China; ^4^Institute of Cardiovascular Sciences and Key Laboratory of Molecular Cardiovascular Sciences, School of Basic Medical Sciences, Ministry of Education, Peking University Health Science Center, Beijing, China

**Keywords:** DSBs, APC7, genome stability, ulcerative colitis, *B. infantis* CGMCC0460.1

## Abstract

Probiotics represents a promising intestinal microbiota-targeted therapeutic method for the treatment of ulcerative colitis (UC). Several lines of evidence implicate that *Bifidobacterium infantis* serves as a probiotic strain with proven efficacy in maintaining the remission of UC. However, the exact mechanisms underlying the beneficial effects of *B. infantis* on UC progression have yet to be elucidated. Herein, we provide evidence that *B. infantis* acts as a key predisposing factor for the maintenance of host genome stability. First, we showed that the fecal microbiota transplantation (FMT) of UC-derived feces contributes to more severely DNA damage in dextran sodium sulfate (DSS)-induced mice likely due to mucosa-associated microbiota alterations, as reflected by the rapid appearance of DNA double strand breaks (DSBs), a typical marker of genome instability. Genomic DNA damage analysis of colon tissues derived from healthy controls, patients with UC or dysplasia, and colitis associated cancer (CAC) patients, revealed an enhanced level of DSBs with aggravation in the degree of the intestinal mucosal lesions. To evaluate whether *B. infantis* modulates the host genome stability, we employed the DSS-induced colitis model and a TNFα-induced intestinal epithelial cell model. Following the administration of C57BL/6 mice with *B. infantis via* oral gavage, we found that the development of DSS-induced colitis in mice was significantly alleviated, in contrast to the colitis model group. Notably, *B. infantis* administration decreased DSB levels in both DSS-induced colitis and TNF-treated colonial cell model. Accordingly, our bioinformatic and functional studies demonstrated that *B. infantis* altered signal pathways involved in ubiquitin-mediated proteolysis, transcriptional misregulation in cancer, and the bacterial invasion of epithelial cells. Mechanistically, *B. infantis* upregulated anaphase-promoting complex subunit 7 (APC7), which was significantly suppressed in colitis condition, to activate the DNA repair pathway and alter the genome stability, while downregulation of APC7 abolished the efficiency of *B. infantis* treatment to induce a decrease in the level of DSBs in TNFα-induced colonial cells. Collectively, our results support that *B. infantis* orchestrates a molecular network involving in APC7 and genome stability, to control UC development at the clinical, biological, and mechanistic levels. Supplying *B. infantis* and targeting its associated pathway will yield valuable insight into the clinical management of UC patients.

## Introduction

Ulcerative colitis (UC), is a chronic inflammatory condition of the colon and is associated with an increased risk for the development of colorectal cancer (CRC). Epidemiological studies demonstrate that more than 20% of worldwide cancers are caused by chronic inflammatory conditions (Grivennikov et al., 2010; Trinchieri, 2012). This increased cancer risk is thought to develop *via* a multistep process involving in cellular genome instability and the progressive accumulation of genetic alterations induced by the chronic inflammation (Loeb and Loeb, 1999). To data, there is no clearly genetic basis explain the alterations in genome stability in patients with UC disease.

Research has demonstrated an increase of higher genomic instability in UC patients, as evidenced by the presence of multiple chromosomal alterations, including DNA double strand breaks (DSBs), DNA replication errors, microsatellite instability (MSI), as well as chromosomal instability, which has been recognized as an early event during the progression of UC-related neoplasia (Willenbucher et al., 1999; Pereira et al., 2016). To further clarify the genetic alterations in UC patients, various of studies depicted the genomic and molecular landscape of inflammatory bowel disease (IBD)-associated CRC, and shown that the main genomic alterations in IBD-CRC, including p53 mutation, p53 loss of heterozygosity (LOH), chromosomic instability, and a high incidence of MSI accompanied by telomere shortening (O’Sullivan et al., 2002), in long standing UC patients with severe inflammation, thus reflecting genomic instability caused by repeated inflammatory stress (Park et al., 1998; Ishitsuka et al., 2001). Notably, continuing chronic inflammation and resultant DNA damage in the setting of UC causes the accumulation of genetic alterations, genomic instability might, therefore, serve as prospective indicators of cancer risk in UC patients. It has been proposed that the anaphase-promoting complex (APC), an E3 ligase enzyme, functions as a tumor suppressor by maintaining genomic stability, whereas the dysregulation of APC/C plays a crucial role in oncogenic processes (Aust et al., 2002; Smolders and Teodoro, 2011). Owing to the heterogeneity of APC subunits expression, the proteins involved in the APC signaling cascade may be altered in UC-related carcinogenesis. As a critical component of APC, the dysregulation of anaphase-promoting complex subunit 7 (APC7) may contribute to the pathogenesis of acute myeloid leukemia (AML) (Rahimi et al., 2015), however, the function roles of APC7 involving in the development of UC remain unclear.

Recent data suggest that microbial factors function as important drivers of inflammation and therefore contribute to carcinogenesis by triggering oxidative stress or changes in genome stability. The high frequency of bacterial cocolonization in colon tissues highlights the importance of understanding the potential effects of gut microbiota in UC development (Guidi et al., 2013). Strong experimental evidence supported the fact that numbers of bacteria that colonized in the intestine could directly affect the genomic integrity of epithelial cells *via* genotoxins. Genotoxin colibactin-producing *pks-Escherichia coli*, was reported to cause cell cycle arrest, DSB, mutations, and promote the development of colitis-associated CRC *in vitro* and *in vivo* (Arthur et al., 2012; Wilson et al., 2019), whereas, *enterotoxigenic Bacteroides fragilis* (ETBF) has been shown to increase the level of interleukin-17 and triggers colon tumorigenesis and DNA damage in the colonic epithelium of Apc Min/+ mice, thus, resulting in faster tumor onset and greater mortality (Wu et al., 2009; Dejea et al., 2018). Thus, it is possible that the intestinal microbiota might affect the ongoing genomic instability of host by direct or indirect ways.

*Bifidobacterium infantis* is a probiotic strain that naturally resides in the human intestines. A striking reduction in the abundance of *B. infantis* has been observed in the intestinal tissue from UC patients (Frank et al., 2007), thus indicating a potential role of *B. infantis* in human intestines. There is also persuasive evidence from several studies to demonstrate that *B. infantis* acted as a probiotic to protect against inflammatory conditions in several ways, including maintaining the balance of gut flora, modulating the immune system, and by producing metabolites that are involved in the amelioration of intestinal inflammation (Peran et al., 2005, 2007; Javed et al., 2016). Despite the strong link between *B. infantis* and inflammatory disease, multiple complementary approaches have provided indirect information with regards to the proven efficacy of *B. infantis* in inducing and maintaining the remission of UC, then limiting our mechanistic understanding of this direct association.

To address this, we performed a comprehensive analysis of histopathology, molecular damage, and gene expression changes, in DSS induced animal models and TNFα-induced colon epithelial cell culture models, that were treated with *B. infantis* or a vehicle. In these models, we were able to reproduce early responses to acute stimulation, including DSBs and inflammation. Furthermore, we examined whether DNA damage caused by DSS or TNFα resulted in genomic instability. In particular, we investigated the signal pathways involved in *B. infantis* treatment, by applying the transcriptional profiling and analysis of GEO database, which indicated that *B. infantis* might upregulate APC7 expression to maintain genome stability and ameliorate inflammation in the development of UC.

## Materials and Methods

### Cell Line and Small Interfering RNAs

Human intestinal epithelial cell lines (HT29 and HCT116) were purchased from ATCC, and maintained in F12 (Gibco, Carlsbad, CA, United States) and RPMI 1640 (Gibco, Carlsbad, CA, United States), supplemented with 10% fetal bovine serum (FBS, Gibco), respectively. All cells were cultured at 37°C and 5% CO_2_. Cells were transiently transfected using Lipofectamine^TM^ 3000 (Thermo Scientific, Worcester, MA, United States). APC7 small interfering RNA (siRNA) and control siRNAs were commercially obtained from Guangzhou RiboBio Co., Ltd. (China). The targeted sequences for si-APC7-1, si-APC7-2, and si-Ctrl were as follows: si-APC7-1: 5′-GGACCAGTATAGTATAGCA-3′; si-APC7-2: 5′-GGAACGCACTGGCTAATCA-3′; si-control: 5′-GGCUCUAGAAAAGCCUAUGCdTdT-3′. The APC7-ovexpressing vectors were purchased from GeneCopoeia, China (#EX-L5377-Lv201).

### Animals

A total of 6–8 week-old male C57BL/6 mice were purchased from Beijing Vital River Laboratory Animal Technology Co., Ltd. (China) and housed under a 12-h light and 12-h dark cycle condition. All laboratory animals were cared for and used in accordance with the guidelines of the Animal Care Ethics and Use Committee of Peking Union Medical College Hospital (Beijing, China).

### Patients and Specimens

All human tissue samples were obtained from endoscopic biopsies or surgical specimens of patients with UC and colitis associated cancer (CAC), in the Peking Union Medical College Hospital between 2015 and 2021. Written informed consent for the application of specimens was obtained from all the patients. The study was approved by the Ethics Committee of Peking Union Medical College Hospital (Beijing, China).

### Administration With *Bifidobacterium infantis* in Dextran Sodium Sulfate-Induced Colitis Model

All mice were initially housed together for adaptive feeding, 1 week later, the mice were randomly divided into control group, colitis model group and *B. infantis* treatment group. The two reference groups (control and colitis model) received sterile saline solution (200 μl/mouse), while the *B. infantis* treatment group received *B. infantis* CGMCC0460.1 powders (7.5 × 10^9^ CFU/ml diluted in 200 μl of sterile saline), purchased from Hangzhou Grand Biologic Pharmaceutical INC. (China). The *B. infantis* treatment group received daily oral administration from day 1 to day 14. Seven days after starting the experiment, 2.5% dextran sodium sulfate (DSS) (Sigma-Aldrich) in drinking water was adequate to induce colitis in the colitis model group and the *B. infantis* treatment group mice for 5 days, and then followed by regulator water for 2 days. Disease activity index (DAI) scores and body weight were recorded throughout the experiments to determine the severity of colitis, as reported previously (Wang et al., 2019). At the end of the experiment, the plasma samples from anesthetized mice was obtained and stored at −80°C. Once the mice were sacrificed, we measured the length of colon and the distal inflamed region of the colon tissues was fixed in 4% buffered paraformaldehyde for histological analysis.

### *Bifidobacterium infantis* Co-cultured With TNFα-Induced Intestinal Epithelial Cells

For *B. infantis* CGMCC0460.1 co-culture, cells at 80% confluency were washed with PBS and incubated in antibiotic-free medium. *B. infantis* CGMCC0460.1 powders were dissolved in antibiotic-free medium, and added to the colon epithelial cells at the indicated concentrations [Multiplicity of Infection (MOI) at 1:100].

### Histological and Immunohistochemical Analysis

Hematoxylin and eosin (H&E) staining was performed on the paraffin-embedded sections from formalin-fixed colonic tissues. Histologic analysis was carried out independently by two pathologists, according to previously validated criteria (Ameho et al., 1997). For IHC analysis, colon sections obtained from patients with UC or CAC, and DSS-induced models were stained with the indicated antibodies, including γH2Ax (CST, Danvers, MA, United States, #9718), p-ATM (Abcam, ab36810) and APC7 (Santa Curze, sc-365649).

### cDNA Synthesis, Genomic DNA Extraction, and Quantitative Real-Time PCR

Total RNA was isolated from indicated colon tissues or cells using TRIzol reagent (Invitrogen), and then subjected to reverse transcription with M-MLV (Promega) in accordance with the manufacturer’s instructions. Quantitative real-time PCR (qRT-PCR) was performed with SYBR Green in an ABI 7500 system (Life Technologies). The primer sequences used for real-time PCR are summarized in Supplementary Table 1, GAPDH was chosen as the internal control for the quantitative analysis. Gene expression levels were determined relative to GAPDH. For the detection of *B. infantis* level, we isolated genomic DNA from indicated feces using Genomic DNA extraction Kit (TIANGEN, Beijing), the concentration of extracted DNA was calculated using a Nanodrop LITE (Thermo Scientific). Then, a total of 25 ng of genomic DNA of each sample was used as a template. Quantification of the total *B. infantis* was performed by qRT-PCR assay, 16 s rRNA was performed as an internal control regarding to the bacteria level quantitation.

### Western Blotting Assay

For western blotting, colon tissues or cell extracts were collected and quantified by the BCA Protein Assay Kit (Thermo Fisher Scientific). Thirty micrograms of each protein sample was resolved by 12% SDS-PAGE (Thermo Fisher Scientific), transferred to PVDF membranes, and then incubated with the indicated primary antibodies at 4°C overnight. Then, the membranes were washed and incubated with secondary anti-rabbit or mouse IgG (CST) antibodies. Subsequently, the binding signals were visualized with an ECL Kit (Pierce Biotech, Thermo Fisher Scientific). GAPDH (Proteintech, HRP-60004) or β-actin (CST, 3700) antibodies was used as an endogenous control.

### Comet Assay

The comet assay was performed using a Comet assay Kit (Trevigen, 4250-050-K), as described previously (Han et al., 2020). In brief, colon epithelial cells were harvested after the indicated treatment, and then mixed with 0.5% low-melting-point agarose, and immediately pipetted onto Cometslide^TM^, which were then placed at 4°C in the dark for 10 min. And, the slides were immersed in 4°C lysis solution for 1 h. Then, the slides were placed in fresh Alkaline Unwinding Solution for 1 h at 4°C, and immersed the slides in Alkaline Electrophoresis Solution for 30 min at 21 volts. After electrophoresis, the slides were gently immersed twice in ddH_2_O for 5 min, and then in 70% ethanol for 5 min, and dried at 37°C for 30 min. Then, the slides were stained with 20 μg/ml of PI (Sigma-Aldrich, St. Louis, MO, United States) and observed under a fluorescence microscope. The comet tails were quantified using CASP software.

### RNA Sequencing

The RNA sequencing was performed by Novogene Co. Ltd. (Beijing, China). Total RNAs were extracted from specimens using TRIzol reagent (Invitrogen, Carlsbad, CA, United States). The concentration of extracted RNA was measured using the Qubit^®^ RNA Assay Kit in a Qubit^®^ 2.0 Fluorometer (Life Technologies, CA, United States). Then, sequencing libraries were generated using the NEBNext^®^ Ultra^TM^ RNA library Prep Kit for Illumina^®^ (NEB, United States). The library preparations were sequenced on an Illumina HiSeq platform. Raw data was firstly processed using in-house perl scripts. The clean data was mapped to the reference genome using STAR (v2.5.1b). The number of reads was counted by HTSeq v 0.6.0 and the Fragments Per Kilobase Million (FPKM) of each gene was calculated to estimate gene expression levels. Gene quantification data were analyzed in R using the DESeq2 package to screen differentially expressed genes. Genes with an adjusted *p*-value < 0.05 and absolute fold change >0, as determined by DESeq2 were considered as significantly differentially expressed.

### Metagenomic Sequencing

Fecal samples were obtained from mice after DSS treatment, then the samples were frozen immediately and underwent DNA extraction using standard protocols. Illumina GAIIX and HiSeq 2000 platform were performed to sequence the samples. Then, clean data was blasted to the host database and were quality filtered using Bowtie 2.2.4 software (Bowtie2.2.4^1^), as described previously (Nielsen et al., 2014).

### Fecal Microbial Transplantation in an Antibiotic-Depletion Model

Mice aged 4–5 weeks were treated with antibiotic and anti-fungal water, in order to deplete the gut microbiota. Briefly, mice were treated with an antibiotic cocktail consisting of 0.5 g/L vancomycin (Sigma-Aldrich), 2 g/L streptomycin (Selleck), 0.75 g/L metronidazole (Sigma-Aldrich), and 0.5 g/L fluconazole (Selleck) for 1 week (Kennedy et al., 2018). Then, the feces were dissolved in 10 g/L sterile saline, 20 μl were cultured on the brain heart infusion (BHI) broth (Biobw, Beijing). The plates were placed in a 37°C incubator, after drying, the plates was inverted and cultured for 24 h under anaerobic condition. The number of colonies on each plate was calculated to evaluate the success of the antibiotic-depletion model. Following the depletion of gut microbiota, fresh stools were collected from the healthy control (healthy volunteers were omnivorous and were not taking antibiotics within 8 weeks before stool collection) or a patient with severe UC (an endoscopic Mayo score of ≥2, and do not use antibiotics or probiotics within 6 weeks before stool collection). Specifically, the stools were dissolved in 10 g/L sterile saline and centrifuged at 2000 rpm for 5 min. The supernatants of stools were administered to the antibiotic-treated mice (200 μl/mice) for 5 consecutive days, then DSS treatment 4 weeks later to induce colitis.

### Statistical Analysis

Statistical analysis was carried out using SPSS version 26.0 software. Data from at least three independent experiments are presented as the means ± SEM. The student’s two-tailed *t*-test or one way analysis of variance (ANOVA) with Tukey’s *post hoc* test were used to determine significant differences among groups. *p*-Value < 0.05 was considered statistically significant. All graphs in this study were prepared using GraphPad Prism version 8.0 software.

## Results

### The Development of Ulcerative Colitis Was Associated With Genome Instability, Which Was Partially Mediated by Alteration in the Gut Microbiota

**FIGURE 1:**
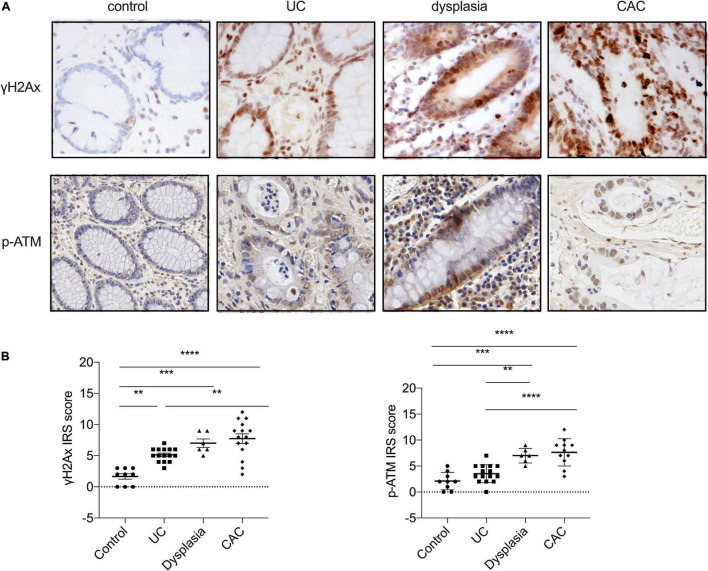
Ulcerative colitis development is associated with genome instability. **(A)** The expression of S139-phosphorylation of histone H2Ax (γH2Ax) and ATM phosphorylation (*p*-ATM) levels were determined by immunohistology assay in healthy controls, patients with UC or dysplasia, and patients with colitis-associated cancer (CAC). (Magnification, ×20.) **(B)** Statistical analysis of γH2Ax and ATM phosphorylation levels determined by immunohistology assay. *p < 0.05, **p < 0.01, ***p < 0.001, ****p < 0.0001.

Since previous studies reported that genome instability is an early event during the progression of UC-related neoplasia (Willenbucher et al., 1999), we aimed to test whether the progression of UC was associated with genome instability, by evaluating the expression of S139-phosphorylation of histone H2Ax (γH2Ax), a sensitive marker of DSBs, using immunohistology. As outlined in Figure 1, γH2Ax foci were localized to the nucleus of epithelial cells, and an increased intensity of γH2Ax foci were seen in biopsies of the colon from UC patients; only very week expression of γH2Ax foci was observed in normal colon tissues. According to the Montreal classification system, the normal colonic mucosa could progress into moderate UC, severe UC, dysplasia, and eventually developed into colitis-associated cancer (Allen and Sears, 2019). Next, we analyzed the relative numbers of γH2Ax foci in different stages of UC progression. Immunohistological analysis showed that the number of positively stained nuclei was significantly higher in inflamed (*p* < 0.05), dysplastic (*p* < 0.001), and CAC tissues (*p* < 0.0001) than that in normal mucosal tissues, thus indicating that γH2Ax expression was sequentially upregulated from normal, UC, dysplasia to CAC. Consistently, the level of ataxia telangiectasia mutated (ATM) phosphorylation was also showed a gradual increase among with aggravation of the degree of the colonic mucosal lesion (Figure 1). These results suggested that genome instability, as indicated by γH2Ax and p-ATM, was involved in the progression of UC.

**FIGURE 2:**
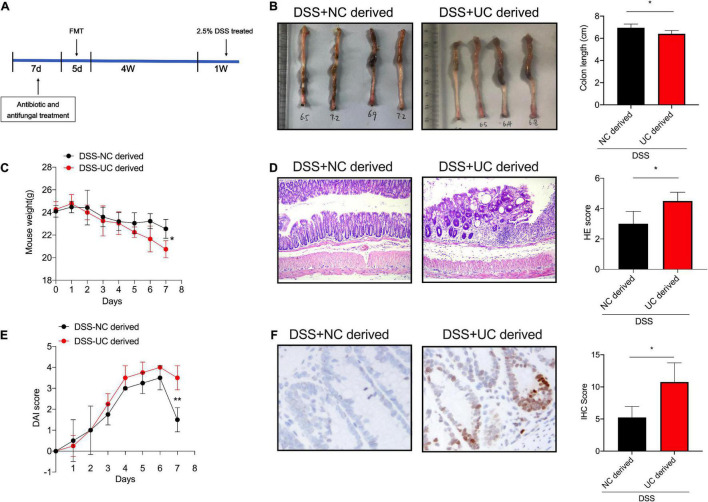
DSS-induced colitis was evaluated among the mice administrated with feces derived from UC patients or healthy controls. **(A)** Schema of FMT model. The colon length **(B)**, body weight **(C)**, the H&E staining of sections **(D)**, and DAI score **(E)**, in colon tissues of mice between the two groups. (Magnification, ×20.) **(F)** The expression levels of γH2Ax were determined by immunohistology in colon tissues of mice administered with feces derived from UC patients and healthy controls. (Magnification, ×20.) *p < 0.05, **p < 0.01.

Indeed, the intestinal microbiome has long been suggested to be involved in UC development and genome stability modulation. Hence, to determine whether the microbiome differences between healthy control and severe UC microbiomes are responsible for the regulation of genome stability in colitis, we performed fecal microbiota transplantation (FMT) assay to determine the influence of microbiota derived from UC patients or healthy controls with regards to the pathogenesis of UC. Firstly, the mice were pretreated with antibiotic cocktail for 1 week to target bacteria and fungal blooms, as the colony formation results suggested, the number of colony derived from antibiotic and antifungal treatment mice was significantly decreased than those from the control mice (Supplementary Figure 1). Then, the microbiomes derived from UC patients or healthy controls were transplanted into the antibiotics-pretreated mice for 5 days. After 4 weeks of colonization to allow microbiota reconstitution in the intestine, we induced colitis by administering 2.5% DSS to mice. We found that feces derived from UC patients aggravated DSS-induced colitis in mice when compared with those derived from healthy controls (Figures 2A–E). Next, immunohistology was performed to investigate the expression levels of γH2Ax. Significant numbers of γH2Ax foci were identified in the nuclei of enterocytes exposed to fecal microbiota derived from UC patients when compared with those from healthy controls, which indicated that gut microbiota is responsible for the genome stability in DSS-induced colitis (Figure 2F).

As previous suggested, the gut microbiomes between healthy controls and UC patients were differently abundant in certain bacterial species. Microbiomes derived from healthy control have increased *Akkermansia*, *Bifidobacterium*, *Lactobacillus*, while those derived from severe UC patients have increased with *Bacteroides, Escherichia-Shigella*, and *Enterococcus* (Sood et al., 2009). In turn, we explored to detect the level of *B. infantis*, belongs to the *Bifidobacterium*, in feces of healthy control and UC patient, and found that *B. infantis* was significantly downregulated in UC patient than those from the healthy control (Supplementary Figure 2). Therefore, our data indicated that genome instability was associated with the development of UC and was partially mediated by alteration of intestinal microbiota.

### The Administration of *Bifidobacterium infantis* Promoted the Recovery of Dextran Sodium Sulfate-Induced Intestinal Injury and Modulated the Composition of Gut Microbiota in Mice

**FIGURE 3:**
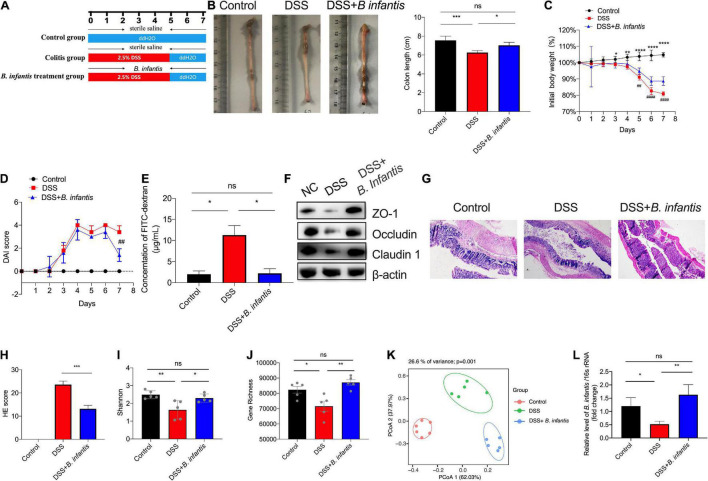
The administration of *B. infantis* relieved colitis in a dextran sodium sulfate (DSS) mouse model of acute colitis. **(A)** Schema of DSS-induced colitis model. Mice in the control group and the colitis model group were pretreated with sterile saline, and the *B. infantis* treatment group was pretreated with *B. infantis* for 7 days. Then, DSS treated mice receiving *B. infantis* by oral gavage as indicated; mice were sacrificed on day 8 and colon length was measured **(B)**. **(C)** Body weight of mice in each group was measured during the experiment period. *Control vs. DSS *p* < 0.05, **Control vs. DSS *p* < 0.01, *****Control vs. DSS *p* < 0.0001, ## Control vs. DSS + *B. infantis p* < 0.01. **(D)** DAI score was monitored each day during the experiment period. ## control vs. DSS + *B. infantis p* < 0.01. **(E)** The FITC assay was performed to determine intestinal permeability. **(F)** The expression levels of tight junction components, including ZO-1, Claduin-1, and Occludin, were detected by western blot assays. H&E staining of sections **(G)** and the total histological score **(H)**. (Magnification, ×20.) **(I–K)** The Shannon index **(I)**, gene richness **(J)** and Principal Coordinates Analysis (PCoA) plot of Bray–Curtis **(K)** regarding to the gut microbiota in DSS-treated models using metagenomic sequencing. **(L)** The level of *B. infantis* in feces of DSS-induced model detected by qRT-PCR assay. Results represent mean ± SEM; *p < 0.05, **p < 0.01, ***p < 0.001, ****p < 0.0001, ns, no significance.

To investigate the effect of *B. infantis* on intestinal colitis, we administered DSS-treated mice with *B. infantis* by oval gavage. The schema of DSS-induced colitis model was presented as in Figure 3A. The mice receiving *B. infantis* presented a milder form of colitis than the mice receiving DSS containing drinking water, as evidenced by the significant lengthening of the colon (Figure 3B). Maximum body weight loss of the mice in the colitis model group was observed on day 7. In contrast, the mice that were given an oral dose of *B. infantis* rapidly recovered weight from day 6 to day 7 (Figure 3C). The DAI score in DSS-treated mice showed an obvious increase on day 7 compared with the control mice, but was downregulated in mice following *B. infantis* administration (Figure 3D). In the colitis model group, the upregulation of DAI score corresponded with increased intestinal permeability, as evidenced by a fluorescein isothiocyanate (FITC) dextran and western blotting assays. However, following exposure to *B. infantis*, the intestinal permeability was decreased and components of the tight junctions, including ZO-1, Claudin-1, and Occludin, were found to be upregulated in the colon by the end of the experiment (Figures 3E,F). In addition, H&E stained sections of colonic tissue showed a significantly higher level of inflammatory cell infiltration and total histological score, along with severe disruption of the mucosal epithelium, in DSS-induced mice, but not in *B. infantis*-treated mice (Figures 3G,H). These results suggested that the administration of *B. infantis* plays a beneficial role in DSS-induced colitis, and helps to alleviate epithelial damage and promote the remission of inflammation.

In addition, we performed metagenomic sequencing to examine the composition of gut microbiota in DSS-induced mice. Although no difference in species richness was measured between the gut microbiotas of DSS-treated mice and the control group, the Shannon index and gene richness were decreased in the DSS-treated group, in contrast to the control group, while following *B. infantis* treatment, the Shannon and gene richness were significantly elevated (Figures 3I–J). PCoA of control, colitis model and *B. infantis* treatment mice from samples indicated significant difference among the three groups (Figure 3K). Subsequently, we detected the level of *B. infantis* in DSS-induced model, and found that *B. infantis* was decreased after DSS treatment, which was partially restored after treatment with *B. infantis* (Figure 3L). Thus, these results indicated that supplementation of *B. infantis* modulated the composition of the gut bacteria, particularly the level of *B. infantis.*

### *Bifidobacterium infantis* Maintained Genome Stability in Dextran Sodium Sulfate-Induced Colitis and TNFα-Treated Intestinal Epithelial Cells

**FIGURE 4:**
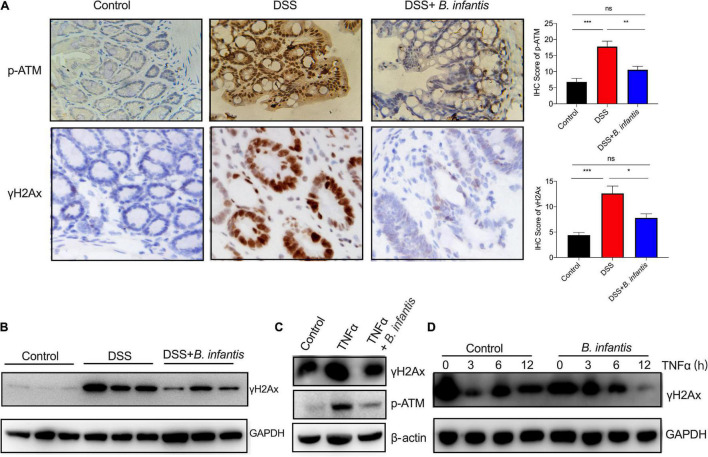
*Bifidobacterium infantis* protected against genome instability in DSS-induced colitis and TNFα-treated intestinal epithelial cells. γH2Ax expression and ATM phosphorylation were determined in DSS-induced mice that had been administered with *B. infantis*, as determined by IHC assays **(A)** and western blotting **(B)**, respectively. (Magnification, ×20.) **(C)** The expression of γH2Ax was examined in TNFα-treated colon epithelial cells that had been co-cultured with *B. infantis*. **(D)** HT29 cells were pretreated with TNFα for 6 h, after which media was replaced with fresh media containing vehicle or *B. infantis* to allow the cells to recover. Western blotting analysis of phosphorylation of γH2Ax was performed. *p < 0.05, **p < 0.01, ***p < 0.001, ns, no significance.

Since gut microbes can elicit their effects on the genome or epigenome *via* direct or indirect mechanisms (Allen and Sears, 2019), we hypothesized that *B. infantis* might exert impact on genome instability triggered by intestinal inflammation. Consistent with this hypothesis, γH2Ax immunohistochemistry and western blot assays revealed significantly enhanced DNA damage in the colon epithelial cells of DSS-induced mice; however, the levels of γH2Ax were obviously decreased after treatment with *B. infantis* (Figures 4A,B). Furthermore, we also investigated the phosphorylation of ATM, a core component of the DNA repair system (Marechal and Zou, 2013). Interestingly, the staining of phosphorylated ATM revealed a similar pattern as seen in the colon tissues with regards to γH2Ax staining (Figures 4,B). These findings show that *B. infantis* exerts impact on DNA damage in intestinal colitis.

To validate the *in vivo* findings observed in DSS-induced mice, we used an *ex vivo* model of cultured-human intestinal cells that were treated with a proinflammatory cytokine (TNFα). When compared with control cells, the levels of phosphorylated H2Ax were significantly higher in TNFα-treated cells (Figures 4C, 6A). Subsequently, we investigated the direct effects of *B. infantis* on the genomic stability of colon epithelial cells. To do this, we developed a co-culture protocol in which *B. infantis* was added into the *ex vivo* model of colon epithelial cells treated with TNFα. Cells were then immunostained for γH2Ax; we observed a notable increase in the expression levels of γH2Ax in cultures treated with TNFα. In contrast, *B. infantis* treatment reduced the amount of DSBs in intestinal cells after cultured for 6 h (Figure 4C). To analyze whether *B. infantis* regulated DSB repairs, we pretreated HT29 cells with TNFα for 6 h, replaced the media with fresh media containing *B. infantis*, and allowed the cells to recover. As shown in Figure 4D, the level of DNA damage gradually reduced in colonial cells after TNFα treatment, thus suggesting the efficient repair of DSBs. In contrast, DNA repair was shown to be advanced in colonial cells that were co-cultured with *B. infantis* (Figure 4D). These data suggest that the increased basal levels of DSBs in TNFα-induced intestinal cells were attenuated by *B. infantis* treatment.

**FIGURE 5:**
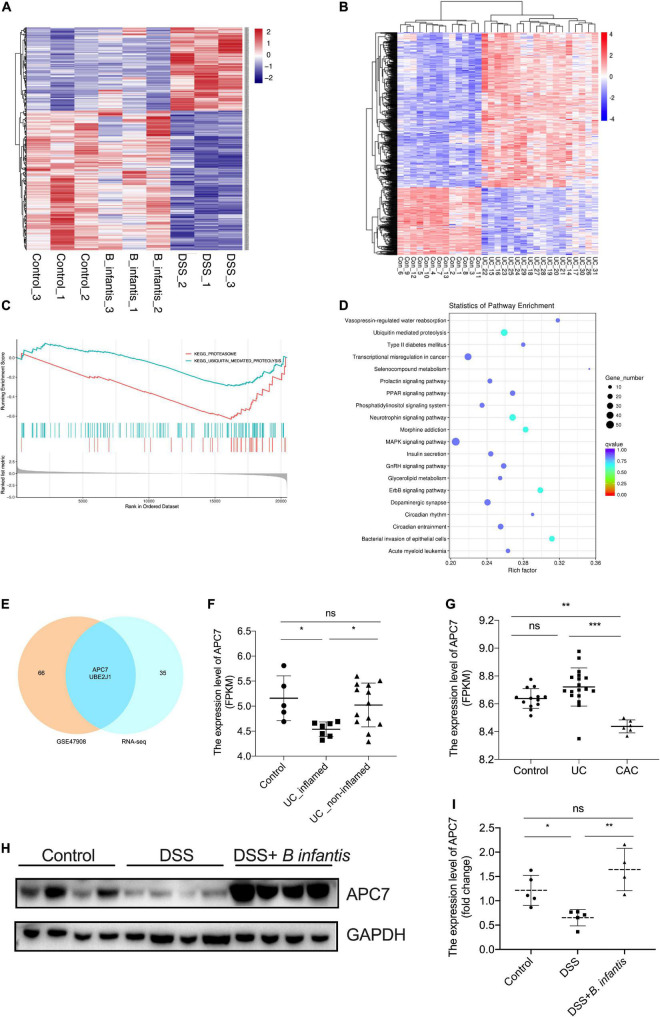
Identification of APC7 as a molecule related to *B. infantis* treatment. **(A)** Heatmap of differentially expressed mRNAs in colon tissues from mice in the control, DSS-treated, and *B. infantis* treatment groups. **(B)** Heatmap of differentially expressed mRNA in colons tissues obtained from healthy controls, patients with UC, from GSE47908. **(C)** GSEA analysis of differentially expressed mRNAs in colon tissues of mice obtained from control, DSS-treated, and *B. infantis* administration groups. **(D)** KEGG analysis of differentially expressed mRNA in colon tissues obtained from healthy controls, patients with UC from GSE47908. **(E)** Venn plot of genes involved in the ubiquitin-mediated proteolysis pathway enriched in RNA-seq results and GSE47908. **(F,G)** The expression level of APC7 were analyzed in colon tissues obtained from healthy controls, patients with UC, and patients with CAC, from GSE47908 and GSE9452. The expression of APC7 was determined using western blotting assays **(H)** and qRT-PCR **(I)** in colon tissues of mice obtained from control, DSS-treated, and *B. infantis* administration groups. *p < 0.05, **p < 0.01, ***p < 0.001, ns, no significance.

**FIGURE 6:**
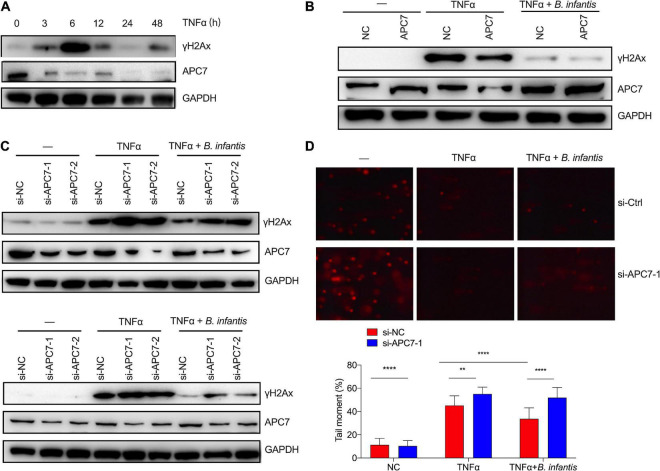
*Bifidobacterium infantis* protected against genome stability partially by upregulating APC7 expression. **(A)** The expression level of APC7 and γH2Ax were determined in TNFα-treated colon cells that had been co-cultured with *B. infantis.* **(B)** γH2Ax levels were investigated by western blotting in APC7-overexpressing HCT116 cells co-cultured with *B. infantis* for 6 h. **(C)** The expression levels of γH2Ax and APC7 were determined in APC7-knockdown colon epithelial cells, including HT29 (top) and HCT116 (bottom), co-cultured with *B. infantis* for 6 h. **(D)** The comet assay was used to determine DNA damage level in APC7-knockdown HT29 cells co-cultured with *B. infantis* for 6 h. (Magnification, ×20.) **p < 0.01, ****p < 0.0001.

### Identification of Anaphase-Promoting Complex Subunit 7 as a Molecule Related to *Bifidobacterium infantis* Treatment

Next, to determine the protective mechanisms underlying the anti-colitis activity of *B. infantis*, we used RNA sequencing to analyze the transcriptome of colon tissue obtained from control, DSS-induced, and *B. infantis*-treated mice (Figure 5A). As the heatmap suggests, there were systematic variations (*p* < 0.05, absolute fold change ≥0) in the expression levels of mRNA related to intestinal colitis (Figure 5A). Gene set enrichment analysis (GSEA) revealed a significant enrichment in genes related to the ubiquitin-mediated proteolysis pathway (Figure 5C). This result was further supported by the transcriptome profiling data from healthy controls, patients with UC in GSE47908 (Figures 5B,D). Then, we screened critical molecules that cause or favor the development of UC, as based on a comparison of the overlapping mRNAs involved in the ubiquitin-mediated proteolysis pathway, enriched in the two RNA-sequencing results (Figure 5E and Supplementary Table 2). Given that the ubiquitin-mediated proteolysis pathway was significantly enriched with regards to the RNA sequencing results, we analyzed the expression of overlapping mRNAs that were enriched in the ubiquitin pathway in public GEO database, and found that APC7 was remarkably downregulated in UC tissues and CAC tissues, compared with normal mucosal tissues in GSE47908 and GSE9452, while there was no significance in APC7 expression between UC tissues and the normal mucosal tissues in GSE47908 (Figures 5F–G). Moreover, we determined the expression of APC7 in colon tissues obtained from DSS-induced colitis by western blotting assays. Comparison between control and DSS-treated tissues shown a marked decrease of APC7 expression level upon DSS treatment, while enhanced following administration with *B. infantis* (Figure 5H). Also, qRT-PCR assay conformed that induction of colitis by DSS led to a significant decrease in the mRNA expression of APC7 in colon tissues when compared to vehicle-treated mice, whereas it upregulated in mice exposed to *B. infantis* in colon tissues; these findings were consistent with the RNA sequencing results (Figure 5I). In conclusion, we speculate that APC7 expression can be affected by *B. infantis* and might be required for the progression of UC.

### *Bifidobacterium infantis* Protected Against Genome Stability by Upregulating Anaphase-Promoting Complex Subunit 7 Expression

To test *in vitro* whether *B. infantis* intervention alters APC7 expression, we first assessed whether APC7 can be affected by TNFα challenge in colon epithelial cells. We found that the expression of APC7 was strongly reduced since TNFα treatment for 3 h, whereas γH2Ax level gradually increased with TNFα treatment and reached peak at 6 h, then returned to the baseline (Figure 6A). Then, the colon epithelial cells were co-cultured with *B. infantis* without inflammation, and the western blot results revealed that *B. infantis* directly upregulated APC7 expression in colon epithelial cells following cocultured with *B. infantis* for 6 h (Supplementary Figure 3).

Previous studies have reported that APC7 plays an important role in the ubiquitin pathway, which was usually related to the maintenance of genome stability (Ghosh and Saha, 2012). Therefore, we investigated whether APC7 played a role in the reduction of DSBs following treatment with *B. infantis*. To characterize the potential effect of APC7 on genome stability in colonial cells, we generated APC7-overexpressing colon epithelial cells using lentivirus-mediated overexpression. As expected, the overexpression of APC7 led to a reduction in the level of γH2Ax in TNFα-induced colonial cells when compared with control cells, as demonstrated by western blotting (Figure 6B). Conversely, the depletion of APC7 abolished the capacity of *B. infantis* treatment to reduce the levels of γH2Ax in TNFα-induced colonial cells, as examined by western blotting assay (Figure 6C). In addition, a neutral comet assay was employed to compare the level of DNA damage in response to APC7 knockdown (Figure 6D). The results also revealed that knockdown of APC7 in colonial cells resulted in longer comet tails than those in the control group upon TNFα treatment, thus indicating that *B. infantis* treatment maintain genome stability partially by upregulating the expression of APC7.

## Discussion

As mentioned previously, widespread genomic instability is known to occur in patients with UC who develop colonic neoplasia, this may, therefore, represent as a marker of cancer risk in UC patients (Itzkowitz and Yio, 2004). Thus, there is a clear need to investigate this mechanism further to explain the genomic instability associated with UC in this process. The non-oncogenic or oncogenic activity of the human gut microbiota has recently become the focus of intense research efforts. However, despite extensive studies on the protective effects of *B. infantis*, there is still no evidence to support the exact mechanisms of underlying the effects of *B. infantis* on the alleviation of colitis. In this study, we demonstrated here APC7, screened by RNA sequencing, may be associated with *B. infantis*-mediated maintain of genome stability and thus might aggravate the progression of UC. Specifically, gut microbiota derived from UC patients promoted genome instability and increased the susceptibility of mice with DSS-induced colitis. With regards to the composition of gut microbiota, our previous studies suggested that an obvious decrease of *B. infantis* was identified in UC patients, and that might play important roles in UC development. In turn, our results supported the fact that *B. infantis* effectively promotes DNA repair by upregulating APC7 expression under colitis conditions.

Patients who suffer from UC symptoms have an increased risk of developing CRC that is thought to develop through genomic instability, thus resulting in the accumulation of genetic alterations that drive tumor progression (Ekbom et al., 1990; Loeb and Loeb, 1999; O’Sullivan et al., 2002; Bartkova et al., 2006; Iftekhar et al., 2021). Previous studies investigated the expression of the DSB marker γH2Ax in patients with longstanding UC, CRC and normal colonic mucosal biopsies and indicted that dysplastic UC was associated with high levels of the DNA damage (De Angelis et al., 2018). Also, oxidative DNA lesions were accompanied with the upregulation of DSB response and repair components in IBD and CAC tissues, indicating that DSBs are the core molecular lesion under inflammatory stress (Frick et al., 2018). Thus, we considered that the grade of colitis inflammation might be associated with the level of genome instability. Here, we evaluated the degree of DNA damage in colon tissues obtained from healthy controls, along with tissues obtained from patients with colitis, dysplasia, and CAC, by γH2Ax immunohistochemistry, and found that the level of γH2Ax was sequential upregulated with increasing grades of inflammation and dysplasia, consistent with previous results (Frick et al., 2018). Nevertheless, the mechanisms of the ongoing genomic instabilities involved in the progression of UC remain poorly understood. There was one explanation mentioned that chromosomal instability in UC might be related to telomere shortening (O’Sullivan et al., 2002). Metagenomic and transcriptomic analysis have revealed that the intestinal microbiome involved in UC development, and that various species of gut microbes have been reported to be altered in stool and mucosal samples from patients with UC (Machiels et al., 2017). Various bacterial strains can produce metabolites or toxins that induce host-cell DNA DSBs and activate of the DNA damage signaling pathway. As a result, when examining the impact of the gut microbiota from UC patients on UC development, we reported that feces, obtained from patients with longstanding severe UC symptoms, were transplanted into the mice that has been pretreated with antibiotics, this resulted enhanced DNA damage in the colon tissues, compared with those from the healthy controls, these results supported that the gut microbiota derived from UC patient is considered as the driving force for genome instability in the development of UC.

Several Gram-negative bacteria, such as *E. coli* and *ETBF*, have been reported to induce DNA damage caused by their secreted toxins that may contribute to the oncogenic process (Guidi et al., 2013; Allen and Sears, 2019). In studies conducted by Dejea et al. (2018), *pks* + *E. coli* were found to work synergistically with ETBF to cause increased DSBs, chromosomal aberrations and cell cycle arrest, *in vitro*. In another study, Yu et al. (2017), showed that, *Fusobacterium nucleatum* promotes chemoresistance *via* the autophagy network to CRC. Together, this evidence points to a direct interaction between specific gut microbes and DNA damage.

Except for the gut microbes mentioned above, there are several beneficial microbes that were remarkedly reduced in the feces of UC patients, including *B. infantis*, *Faecalibacterium*, and *Lactobacillus* (El-Baz et al., 2020; Nishihara et al., 2021). Our previous results also confirmed that the amount of *B. infantis* was reduced upon colitis inflammation (Wang et al., 2019). With the increasing number of reports on the roles of *B. infantis*, there has been significant interest in investigating the specific mechanism of action that is involved. We aimed to explore whether *B. infantis* impact the genome stability. The administration of DSS-induced mice with *B. infantis* could promote an ATM-dependent DNA damage response that was characterized by the formation of γH2Ax foci in the nucleus of colon epithelial cells. Additionally, we also used a TNFα-treated colonial cell model to evaluate the functional roles of *B. infantis in vitro* study. Our results support the importance of *B. infantis* in colitis by modulating DNA damage repair. Accordingly, we dissected the underlying mechanisms by which *B. infantis* treatment maintains genomic stability by applying RNA sequencing and public database analysis. Our results showed that in response to colitis, epithelial APC7 was increased by *B. infantis* administration, this was found to be dysregulated in the colon of UC patients. APC7, one component of the APC with E3 ubiquitin ligase activity, controls mitotic cell cycle progression. The timely activation of APC is thought to be important to maintain accurate chromosome separation, whereas the dysregulation of APC may give rise to abnormal chromosome segregation, then leading to chromosome instability (Sudakin et al., 2001; Greil et al., 2016). Considering that APC is required to maintain genomic stability in primary human cells (Engelbert et al., 2008), we detected whether APC was associated with the effect of *B. infantis*-treatment on maintaining of genomic stability. Our experiments indicated that overexpression of APC7 enhanced DNA genomic stability in TNFα-treated colon epithelial cells, while APC7 knockdown promoted DNA damage upon co-culture with *B. infantis*. However, future research should clarify whether this is also the case *in vivo*, and in patients treated with *B. infantis* strains.

## Conclusion

**FIGURE 7:**
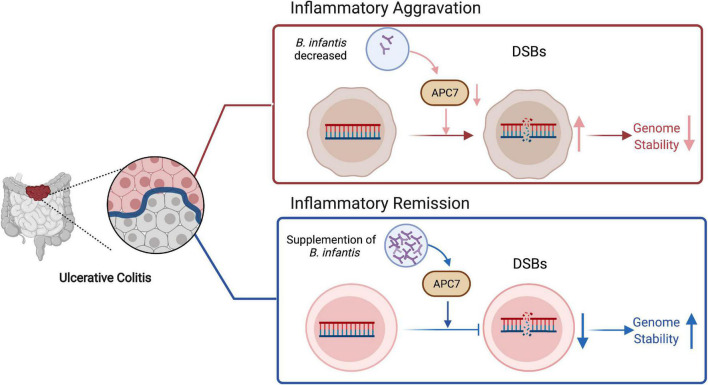
Proposed work model for *B. infantis* to regulate genome stability through upregulating of APC7 expression in colitis, created with BioRender.com.

In summary, these data suggest that genomic instability is associated with UC development. In this process, co-colonization with *B. infantis* strains enhanced genomic stability by increasing APC7 expression in colon tissues, therefore alleviating colitis inflammation (Figure 7). This study implies that the evaluation of probiotic strains, could reduce the risk of colitis related cancer.

## Data Availability Statement

The data presented in the study are deposited in the https://bigd.big.ac.cn/gsa/browse/CRA005171; repository, accession number CRA005171.

## Ethics Statement

The studies involving human participants were reviewed and approved by the Ethics Committee of Peking Union Medical College Hospital (Beijing, China). The patients/participants provided their written informed consent to participate in this study. The animal study was reviewed and approved by the Ethics Committee of Peking Union Medical College Hospital, Beijing, China (protocol code: XHDW-2019-031).

## Author Contributions

TH, XH, and JL were involved in the overall design of the experiments. TH, XH, KL, and DZ participated in the experiments. TH and XH wrote the manuscript. YZ and JL assessed and interpreted the results. All authors have read and approved the published version of the manuscript.

## Conflict of Interest

The authors declare that the research was conducted in the absence of any commercial or financial relationships that could be construed as a potential conflict of interest.

## Publisher’s Note

All claims expressed in this article are solely those of the authors and do not necessarily represent those of their affiliated organizations, or those of the publisher, the editors and the reviewers. Any product that may be evaluated in this article, or claim that may be made by its manufacturer, is not guaranteed or endorsed by the publisher.

## Footnotes

1
http://bowtie-bio.sourceforge.net/bowtie2/index.shtml

## Supplementary Material

Supplementary Figure 1 Evaluation of antibiotic-depletion model. **(A)** Represent plates of colonies derived from the healthy control and the UC patient. **(B)** Evaluation of antibiotic-depletion model by calculating the colony formation units (CFU) in each plate. **p* < 0.05.

Supplementary Figure 2 The levels of *B. infantis* in feces derived from the healthy control and UC patient, detected by qRT-PCR assay. ***p* < 0.01.

Supplementary Figure 3 The expression levels of APC7 in colon epithelial cells co-cultured with *B. infantis* for 6, 12, 24, and 48 h, respectively.
