# Supplementary figures and images for: Bifidobacterium infantis Maintains Genome Stability in Ulcerative Colitis via Regulating Anaphase-Promoting Complex Subunit 7
Source: Front Microbiol. 2021 Nov 2;12:761113. doi: 10.3389/fmicb.2021.761113 (PMC8593188; doi:10.3389/fmicb.2021.761113)

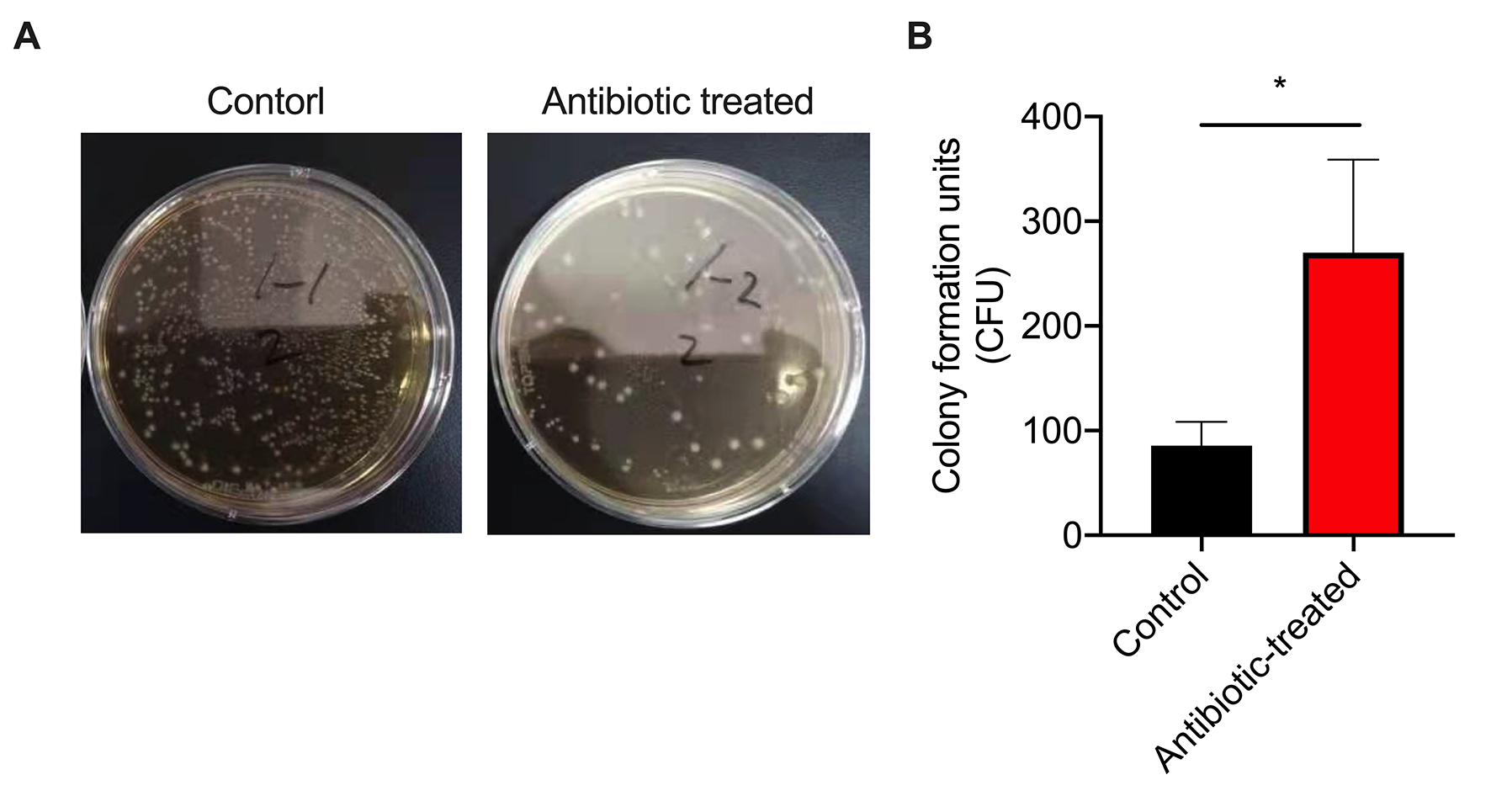

Supplement: Supplementary Figure 1 — Evaluation of antibiotic-depletion model. (A) Represent plates of colonies derived from the healthy control and the UC patient. (B) Evaluation of antibiotic-depletion model by calculating the colony formation units (CFU) in each plate. *p < 0.05. [file Image_1.tiff]

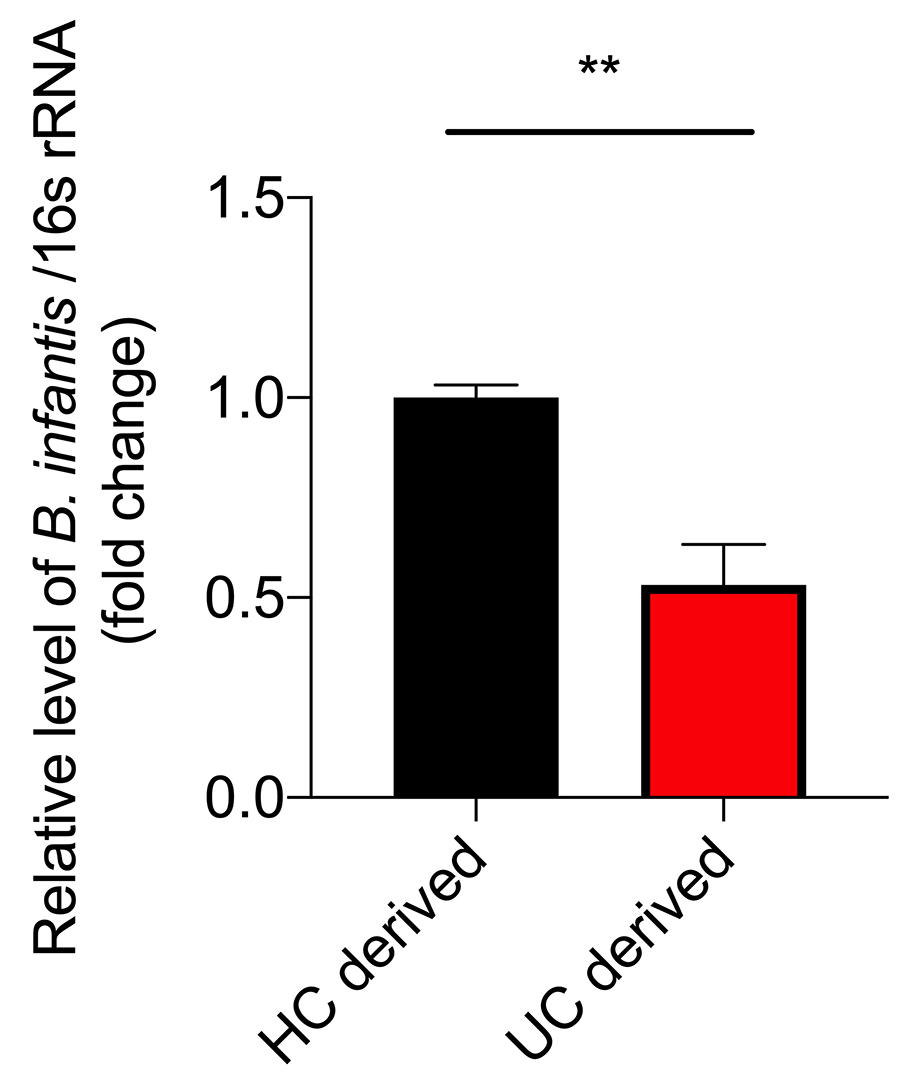

Supplement: Supplementary Figure 2 — The levels of B. infantis in feces derived from the healthy control and UC patient, detected by qRT-PCR assay. **p < 0.01. [file Image_2.tiff]

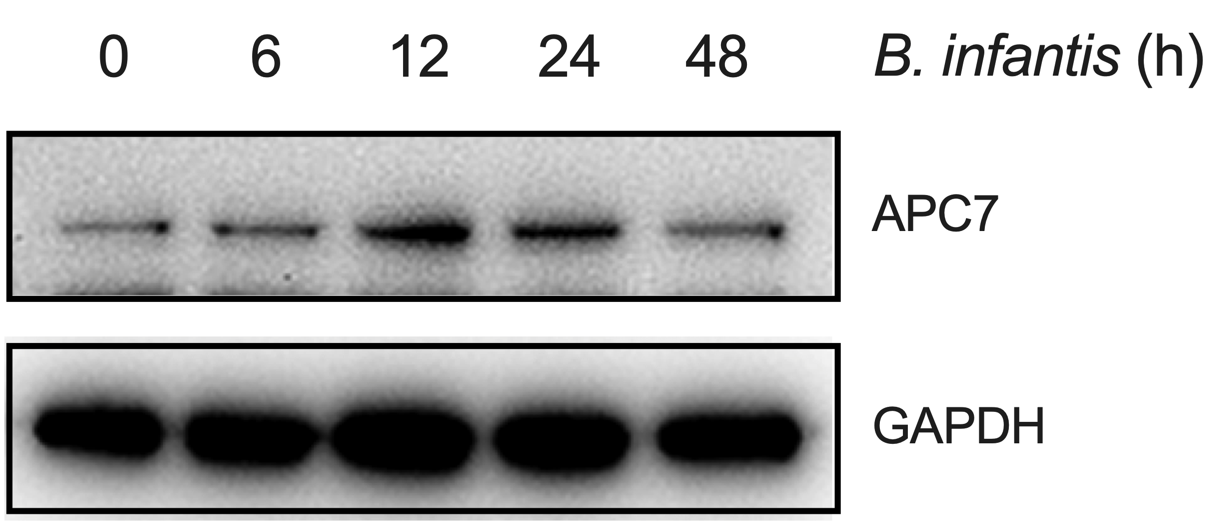

Supplement: Supplementary Figure 3 — The expression levels of APC7 in colon epithelial cells co-cultured with B. infantis for 6, 12, 24, and 48 h, respectively. [file Image_3.tiff]
