# Supplementary material for: Bifidobacterium infantis Maintains Genome Stability in Ulcerative Colitis via Regulating Anaphase-Promoting Complex Subunit 7
Source: Front Microbiol. 2021 Nov 2;12:761113. doi: 10.3389/fmicb.2021.761113 (PMC8593188; doi:10.3389/fmicb.2021.761113)
Supplement: Supplementary file 4 [file Data_Sheet_1.docx]

Supplementary Material

# Supplementary Tables

# Table S1 The primers sequence used to perform qRT-PCR assay.

| **Gene primers** | **Forward** **primer sequence (5’-3’)** | **Reverse primer sequence (5’-3’)** |
| --- | --- | --- |
| GAPDH | ACCACAGTCCATGCCATCAC | TCCACCACCCTGTTGCTGTA |
| APC7 | CTGCCGCCTCTTCAACAT | GCCTCCCGAAAGTGTATG |
| *B. infantis* | CCATCTCTGGGATCGTCGG | TATCGGGGAGCAAGCGTGA |
| 16s | TCCTACGGGAGGCAGCAGT | GGACTACCAGGGTATCTAATCCTGTT |

**Table S2.** Differentially expressed genes involved in the ubiquitin-medicated proteolysis pathway, enriched in RNA-seq results and GSE47908.

| **GSE47908 (n=68)** | **RNA-seq (n=37)** | |
| --- | --- | --- |
| MTA1 | Socs3 | |
| UBE2R2 | Rhobtb1 | |
| UBXN4 | Ube2l3 | |
| ZNRF1 | Ube2f | |
| TMUB1 | Elob | |
| MAN1B1 | Ube2c | |
| FBXL17 | Socs1 | |
| KCTD13 | Cdc34 | |
| ANKIB1 | Klhl13 | |
| DNAJB2 | Ube2m | |
| PCBP2 | Cblc | |
| UBXN1 | Ube2s | |
| CCAR2 | Fzr1 | |
| SPSB3 | Brca1 | |
| RNF187 | Skp1a | |
| AMFR | Nhlrc1 | |
| NPLOC4 | Ube2a | |
| N4BP1 | Anapc11 | |
| FBXL12 | Ube2n | |
| TMUB2 | Cdc20 | |
| WAC | Ube2j2 | |
| TBL1X | Eloc | |
| SMAD7 | Prkn | |
| FBXW4 | Rchy1 | |
| STUB1 | Trim32 | |
| CUL4B | Fbxo4 | |
| FBXO31 | Uba3 | |
| SIAH3 | Map3k1 | |
| MAP1A | Skp2 | |
| FBXL21P | Fbxo2 | |
| ZFAND2B | Uba1 | |
| RNF185 | Ube2k | |
| FBXL15 | Rnf7 | |
| PSME3 | Anapc7 | |
| PSMD11 | Ddb2 | |
| HSP90AB1 | Siah1b | |
| FBXO22 | Ube2j1 | |
| UFD1 |  |  |
| USP5 |  |  |
| PSMC2 |  |  |
| UBE2D3 |  |  |
| EDEM3 |  |  |
| PSME4 |  |  |
| UBC |  |  |
| SOCS5 |  |  |
| CDC27 |  |  |
| USP14 |  |  |
| ANAPC2 |  |  |
| WWP1 |  |  |
| RNF34 |  |  |
| PSMD14 |  |  |
| HSPA5 |  |  |
| PSMA3 |  |  |
| UBE2J1 |  |  |
| TLK2 |  |  |
| HSP90B1 |  |  |
| ANAPC7 |  |  |
| UBXN8 |  |  |
| DDB1 |  |  |
| VCP |  |  |
| UBE2G1 |  |  |
| CHFR |  |  |
| PSMC4 |  |  |
| PSMC6 |  |  |
| PLAA |  |  |
| SDCBP |  |  |
| PSMD7 |  |  |
| SDCBP |  |  |
| FAF2 |  |  |
